# Self‐Reported Adherence to Vegetarian and Vegan Diets: Insights From the 3rd Bavarian Food Consumption Survey

**DOI:** 10.1111/nbu.70029

**Published:** 2025-09-09

**Authors:** Sebastian Gimpfl, Florian Rohm, Nina Wawro, Nadine Ohlhaut, Christine Röger, Melanie Senger, Martin Kussmann, Jakob Linseisen, Kurt Gedrich

**Affiliations:** ^1^ ZIEL—Institute for Food and Health, AG Public Health Nutrition Technical University of Munich Munich Germany; ^2^ Chair of Epidemiology University of Augsburg Augsburg Germany; ^3^ Institute of Epidemiology Helmholtz Centre Munich Munich Germany; ^4^ Competence Center for Nutrition (KErn) Bavarian Research Institution for Agriculture (LfL) Freising Germany; ^5^ Institute for Medical Information Processing, Biometry and Epidemiology, Medical Faculty Ludwig‐Maximilians‐University of Munich Munich Germany

**Keywords:** BVS III, eating habits, Germany, humans, nutrition assessment, nutritional intake, vegans, vegetarians

## Abstract

Vegetarian and vegan diets are increasingly popular in Germany due to ethical considerations, perceived health and environmental benefits. Regionally representative data, particularly for Bavaria, remain scarce. This study updates the prevalence, demographics and eating motives of vegetarians and vegans using data from the 3rd Bavarian Food Consumption Survey (BVS III; 2021–2023), a repeated, population‐based, representative study. Stratified random sampling recruited 1503 adults aged 18–75 years via resident registration offices. Dietary intake was assessed using repeated 24‐h recalls. Participants self‐identified their diets. Individuals indicating ‘vegetarian’ or ‘vegan’ were pooled and compared to omnivores. The prevalence of vegetarian/vegan diets increased from 2.2% (2002/2003) to 6.3%. Higher education (OR: 4.2; 95% CI: 1.7–10.2) and being female (OR: 2.3; 95% CI: 1.2–4.2) significantly predicted adherence, while urbanity and age did not. Compared to omnivores, vegetarians/vegans reported stronger motivations related to health and environmental concerns (*p* < 0.001) but placed less importance on sociability (*p* = 0.017) and traditional eating (*p* = 0.042). Adjusted mean protein intake was significantly lower in vegetarians/vegans (62.4 g/d vs. 70.3 g/d, *p* = 0.004), yet still adequate. Essential amino acid intake was also lower; their relative proportion (~50%) was comparable between groups. Fibre intake was significantly higher among vegetarians/vegans (23.8 g/day vs. 16.5 g/day, *p* < 0.001). The prevalence of vegetarian and vegan diets nearly tripled over two decades. The findings substantiate a regressive trend in meat consumption in the region, driven by health rather than environmental concerns.

## 1 Introduction

Nutrition impacts physical, mental and social well‐being through the communal aspects of food intake and preparation, as well as psychosocial influences on eating behaviours. Dietary behaviours continuously adapt to social, economic, political and cultural changes (Fischler 1988). The growing prevalence of overweight and obesity (Cena and Calder 2020; Trnovec et al. 2001), the emphasis on disease prevention and health promotion (Nutbeam 2019), increasing awareness of environmental sustainability, and the availability of nutrition information are significant factors shaping dietary behaviours (Denniss et al. 2023; Klassen et al. 2018; Ni Mhurchu et al. 2018). Such a shift is reflected in the rising popularity of vegetarian and vegan diets (Leitzmann 2014). Vegetarianism and veganism are dietary types characterised by varying degrees of the omission of animal‐derived foods, including seafood and fish. The term vegetarianism is usually used to describe ovo‐lacto‐vegetarianism, in which eggs and dairy are permitted. However, vegetarianism encompasses several other subcategories, such as lacto‐vegetarianism (permits dairy but excludes eggs), ovo‐vegetarianism (permits eggs but excludes dairy) and veganism (Leitzmann 2014; Mensink et al. 2016; Richter et al. 2016). Veganism is also emblematically described as strict vegetarianism because it excludes all animal‐derived products, including meat, seafood, fish, dairy, eggs, arguably honey and often non‐food items like leather and wool (Leitzmann 2014). While these definitions appear straightforward, there is ongoing debate in the scientific and public discourse regarding their boundaries and classification (Hargreaves et al. 2023). Some variations, such as flexitarianism, semi‐vegetarianism and pescatarianism (also called pesco‐vegetarianism), which involve occasional meat or fish consumption, lack consensus and blur the lines. Furthermore, individuals identifying themselves as vegetarians may occasionally still consume animal‐derived foods and challenge a strict definition (Rosenfeld 2018). The reasons for following vegetarian and vegan diets are multifaceted and often concurrent. Leitzmann (2014) as well as Leitzmann and Keller (2020) classify these reasons into four main categories: ethical, religious, health‐related and ecological. Ethical motives primarily relate to animal rights, welfare and a rejection of (industrialised) animal farming. In many cases, these ethical concerns align with spiritual/religious beliefs advocating for non‐violence and compassion toward living beings. Health‐related motivations focus on perceived health benefits, including weight control, disease prevention and enhanced physical and mental well‐being. Evidence from several meta‐analyses suggests that adherence to vegetarian or vegan diets is associated with beneficial health outcomes (Dinu et al. 2017; Dybvik et al. 2023; Lv et al. 2025; Ocagli et al. 2023; Yokoyama et al. 2014). Large cohort studies, such as the Adventist Health Study‐2 (AHS‐2) and EPIC‐Oxford, partially support these associations. In these cohorts, vegetarian and vegan dietary patterns were linked to a reduced risk of several chronic diseases, including type 2 diabetes (Orlich and Fraser 2014; Papier et al. 2019), hypertension (Key et al. 2022; Orlich and Fraser 2014) and certain cancers (Key et al. 2022; Tantamango‐Bartley et al. 2013). However, all‐cause mortality rates did not differ between vegetarians, vegans and omnivores in AHS‐2 and EPIC‐Oxford (Key et al. 2022; Orlich and Fraser 2014). Some adverse outcomes have also been observed. In the EPIC‐Oxford study, vegetarians (and especially vegans) showed a higher risk of total and particular hip fractures, and vegetarians also had an increased risk of stroke, largely driven by a higher haemorrhagic stroke risk (Key et al. 2022). They also reported that lower levels of critical nutrients such as vitamin B12, vitamin D and calcium were evident (Key et al. 2022). While median intakes of these nutrients in AHS‐2 were above minimum requirements, the lower ends of the distributions remained concerning (Rizzo et al. 2013), underscoring the importance of dietary attentiveness to ensure adequacy. These assessments were made against Dietary Reference Intakes (Food and Nutrition Board, Institute of Medicine, and National Academies 2012), established for U.S. and Canadian populations, with adult requirements set at 2.0 μg/day for vitamin B12, 10.0 μg/day for vitamin D and 800–1100 mg/day for calcium. In AHS‐2, the 5th percentile intake for strict vegetarians was as low as 0.4 μg/day for vitamin B12, 0.1 μg/day for vitamin D and 520 mg/day for calcium. Median calcium intake (933 mg/day) was closer to recommended levels (Rizzo et al. 2013) but may still fall short, especially for older adults and post‐menopausal women with higher needs. By contrast, dietary vitamin D intake is of limited interpretability, since cutaneous synthesis through UV exposure contributes substantially to overall status.

Ecological motivations are driven by the desire to improve resource efficiency and reduce the environmental impact, such as lower greenhouse gas emissions, conservation of water and reduced land use. The EAT‐Lancet Commission synthesised current scientific evidence to propose a universal reference diet that is both health‐promoting and environmentally sustainable. This reference diet emphasises plant‐derived foods such as whole grains, fruits, vegetables, legumes and nuts. Animal‐derived foods are recommended only in modest amounts or can be entirely omitted, but they have been criticised as not providing sufficient micronutrients (Beal et al. 2023). This approach aligns with global targets for climate change mitigation, biodiversity preservation and sustainable resource use. This expert consensus underscores the role of plant‐based diets as a cornerstone of sustainable nutrition strategies, with relevance to both public health and planetary boundaries (Willett et al. 2019). The lower environmental impact of these diets is supported by numerous studies (Aleksandrowicz et al. 2016; Chai et al. 2019; Gimpfl et al. 2025; Scarborough et al. 2023). The Academy of Nutrition and Dietetics (AND) acknowledges the health benefits of well‐planned vegetarian and vegan diets in their position paper published in 2016. According to the AND, these diets are nutritionally adequate for all stages of life (including pregnancy, lactation, infancy and adolescence), associated with positive health outcomes and environmentally sustainable (Melina et al. 2016). Similarly, the German Nutrition Society (German: *Deutsche Gesellschaft für Ernährung*, DGE) supports the health and environmental benefits while advising caution regarding strict veganism, particularly for vulnerable groups such as pregnant women and children (Klug et al. 2024; Richter et al. 2016). Both organisations highlight the need for careful planning to ensure sufficient intake of critical nutrients, particularly vitamin B12, iron, calcium, vitamin D, omega‐3 fatty acids, zinc and iodine (Klug et al. 2024; Melina et al. 2016; Richter et al. 2016).

According to data on the meat supply in Germany published by the Federal Ministry of Food and Agriculture (German: *Bundesministerium für Ernährung und Landwirtschaft*, BMEL), the calculated per capita meat consumption has gradually declined since 1991 (BMEL 2025). Estimates of the prevalence of vegetarianism in Germany over the past decades have varied between 2% and 12%. Despite national estimates, representative and up‐to‐date data, especially for specific regions such as Bavaria, are missing. In 2006, the Second German National Nutrition Survey (NVS II) indicated that 1.6% of the population aged 14–80 years were vegetarians (Max Rubner‐Institut 2008). The German Health Interview and Examination Survey for Adults (DEGS1) conducted between 2008 and 2011 estimated that 4.3% of the population aged 18–79 years followed a vegetarian diet (Mensink et al. 2016), with neither study distinguishing between vegetarianism and veganism (Max Rubner‐Institut 2008; Mensink et al. 2016). The BMEL publishes annual reports on the German diet based on surveys of 1000 German residents aged 14 years and older. Recently (2021–2023), the proportion of respondents following a vegetarian diet fluctuated between 7% and 10%, while 1%–2% were vegan (BMEL 2021, 2022, 2023). In Bavaria, the 2nd Bavarian Food Consumption Survey II (BVS II) in 2002/2003 reported that 2.2% of the Bavarian population between 13 and 80 years was vegetarian (including vegans) (Karg et al. 2004).

To update the current understanding of dietary patterns in Bavaria, the 3rd Bavarian Food Consumption Survey (BVS III) was conducted from October 2021 to January 2023, assessing dietary intake along with lifestyle and health aspects of the Bavarian population aged 18–75 years. Despite increasing interest in plant‐based diets, particularly vegetarianism and veganism, and their potential health and ecological benefits, up‐to‐date regional data on the prevalence and demographic distribution of vegetarian and vegan diets in Bavaria are lacking. Moreover, the motivational drivers behind these dietary choices remain underexplored, particularly in comparison to omnivorous diets. Therefore, the aim of this work is to describe the pooled prevalence of vegetarianism and veganism across sex, age groups, BMI categories and educational status in Bavaria. Additionally, it seeks to elaborate on drivers that promote adherence and compare eating motives between vegetarians/vegans and omnivores.

## 2 Methods

### 2.1 Study Design

The BVS III was a representative survey conducted in Bavaria, Germany, between October 2021 and January 2023. The target population comprised the general population in Bavaria aged 18–75 years living in Bavaria and in private households with sufficient proficiency in German. Using a stratified, multi‐stage random sampling method, the survey recruited 1503 participants in total. In the first sampling stage, municipalities were randomly selected with replacement to acquire the sample points, stratified by district, province and region type. Addresses were requested from the residents' registration offices. Per sample point, individuals were randomly drawn and asked to participate. Reasons for non‐participation were documented and are described elsewhere (Gimpfl et al. 2025). Participants were first visited at home for a personal interview, questionnaire, anthropometric measurements, and the collection of blood samples to assess various health metrics. After the home visits, participants were asked to complete three 24‐h dietary recalls over 6 weeks by telephone interview using the GloboDiet software to document food consumption. The recalls were ideally conducted on two weekdays and one weekend day; while consecutive days were possible—especially for weekdays—three consecutive days were never scheduled. The assessed food items were matched with the German Nutrient Database [BLS 3.02; German: *Bundeslebensmittelschlüssel 3.02* (Max Rubner‐Institut 2014)]. Comprehensive quality control ensured data accuracy, and the findings were weighted to ensure representativeness based on factors like region, education, sex and age. The reference was the German 2020 microcensus, that is, an extrapolation of the data for Bavaria (Bayerisches Landesamt für Statistik 2023).

**FIGURE 1:**
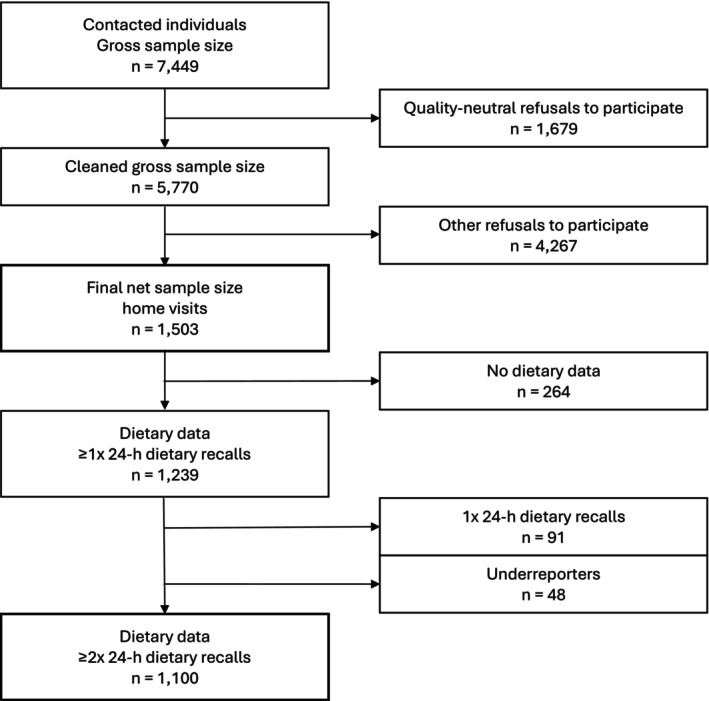
Flow chart of participant recruitment and sample selection for the BVS III. Of the 7449 individuals initially contacted, 1503 completed the house visits, that is, the questionnaires. After sample cleaning, including exclusion of participants with fewer than two 24‐h recalls and under reporters, the final analytical sample for dietary assessment in this work comprised 1100 participants. Relevant sample sizes considered for this study are highlighted with thicker lines.

For this paper, only participants who completed at least two 24‐h dietary recalls were included in food, energy, and nutrient intake analyses, excluding under‐reporters (*n* = 1100). In addition, the total study population was used to analyse questionnaire‐based variables from the home visits (*n* = 1503). A flow chart for the study recruitment is shown in Figure 1. The study design and conduction, including recruitment and response, are described in detail elsewhere (Gimpfl et al. 2025; Rohm et al. 2025).

### 2.2 Measures

The data used in this study included information on participants' sex, age, reported body height and weight, measured waist circumference, education level, smoking habits, physical activity and political municipality category reflecting rural vs. urban differences, in addition to the dietary data. Physical activity was classified according to Gerrior et al. (2006) and EHIS‐PAQ (European Health Interview Survey—Physical Activity Questionnaire) (Finger et al. 2015). During the face‐to‐face interview, participants were asked to self‐identify their diet type by selecting from predefined options: ‘vegetarian’, ‘vegan’, ‘no special diet’ or ‘other’. This classification was based solely on participants' self‐report, with general definitions provided by the interviewer to support understanding. Participants who selected ‘no special diet’ or ‘other’ were categorised as ‘omnivorous’. To ensure sufficient statistical power and meaningful interpretation, the low prevalence of individuals identifying themselves as ‘vegetarian’ or ‘vegan’ were combined into a single ‘vegetarian/vegan’ subgroup, if not explicitly stated otherwise. Participants completed the German version of the Single‐Item Eating Motives questionnaire (Wahl et al. 2020) and were asked to rank each of 15 given motives (liking/appetite, habit, need and hunger, health, convenience, pleasure, traditional eating, natural/environmental concerns, sociability, price, visual appeal, weight control, affect regulation, social norms and social image) on a Likert scale ranging from one to four with increasing degree of agreement (Renner et al. 2012). The questions were preceded by the phrase ‘I eat, what I eat…’ [German (original): ‘*Ich esse das, was ich esse…*’].

### 2.3 Statistical Analysis

Under reporters were defined as having a ratio of the calculated total energy intake to the calculated basal metabolic rate of less than 0.6 and were subsequently excluded from analysis (Figure 1). The basal metabolic rate was calculated according to formulae published by the World Health Organization (WHO) (1985).

Descriptive statistics were used to portray the response and demographic characteristics of the study sample and the Bavarian population. Standard deviations (SD) or standard errors (SE) were calculated for unweighted or weighted data, respectively. 95% confidence intervals (CI) were calculated. Two‐group comparisons were conducted using Wilcoxon's rank‐sum test, with adjustments for survey samples when analysing weighted data. Categorical variables were compared using the chi‐squared test, applying Rao & Scott's second‐order correction for weighted data and Pearson's chi‐squared test or Fisher's exact test for unweighted data. Linear trend analyses were performed using generalised linear models with ordered factors. Odds ratios (OR) were calculated through binary logistic regression, adjusting for sex, age group, education and political municipality category. Energy and nutrient intakes were adjusted for sex and age groups through linear regression. Food group intakes, however, could not be adjusted using regression analyses due to the left‐censored nature of the data; instead, they were energy‐adjusted to 2000 kcal. *p*‐Values below 0.05 were considered statistically significant.

The statistical software R version 4.4.0 was used for all statistical analyses and graphical depictions (R Core Team 2024).

## 3 Results

### 3.1 Demographic Characteristics

The total study population included 1503 participants, 46% male and 54% female. The average age was 48.0 years (SD: 15.1 years). Education, civil status and employment differed by sex (*p* < 0.001), with more males with higher education and full employment (Table 1). Dietary data was available for 1100 participants with at least two 24‐h dietary recalls, excluding under reporters. Characteristics were comparable to the total study population (Table S1). Following, weighted data were used to ensure the representativeness of the Bavarian population (Table 2 and Table S2).

**TABLE 1:** Description of the full BVS III study sample stratified by sex.

Variable	*n*	Overall, *n* = 1503 (100%)^a^	Male, *n* = 688 (46%)^a^	Female, *n* = 815 (54%)^a^	*p‐Value* ^b^
Age (in years)	1503	48.0 ± 15.1	47.7 ± 15.3	48.3 ± 14.9	0.484
Age group (in years)	1503				> 0.9
18–24		109 (7%)	50 (7%)	59 (7%)	
25–34		239 (16%)	115 (17%)	124 (15%)	
35–50		436 (29%)	193 (28%)	243 (30%)	
51–64		479 (32%)	220 (32%)	259 (32%)	
≥ 65		240 (16%)	110 (16%)	130 (16%)	
Education	1502				0.004
Low		339 (23%)	162 (24%)	177 (22%)	
Middle		435 (29%)	170 (25%)	265 (33%)	
High		728 (48%)	355 (52%)	373 (46%)	
Civil status	1501				< 0.001
Single		221 (15%)	114 (17%)	107 (13%)	
Unmarried—in a partnership		212 (14%)	103 (15%)	109 (13%)	
Married		922 (61%)	427 (62%)	495 (61%)	
Widowed		38 (3%)	5 (1%)	33 (4%)	
Divorced		108 (7%)	38 (6%)	70 (9%)	
Living situation	1503				0.344
Living alone in a private household		240 (16%)	108 (16%)	132 (16%)	
Living in a private household with family/friends or other persons		1247 (83%)	570 (83%)	677 (83%)	
Community‐oriented living arrangement		5 (0%)	2 (0%)	3 (0%)	
Other		11 (1%)	8 (1%)	3 (0%)	
Employment	1502				< 0.001
Employed		988 (66%)	475 (69%)	513 (63%)	
Marginally, occasionally or irregularly employed		42 (3%)	8 (1%)	34 (4%)	
In vocational training/apprenticeship/retraining		22 (1%)	14 (2%)	8 (1%)	
Currently not employed: unemployed or job‐seeking, on parental leave		84 (6%)	28 (4%)	56 (7%)	
Retired, pensioner, homemaker		310 (21%)	136 (20%)	174 (21%)	
Other (e.g., pupil, student, assisting family member)		56 (4%)	26 (4%)	30 (4%)	

*Note:* Data are presented as absolute (*n*) and relative frequencies (%) for categorical variables. Age is displayed as mean ± standard deviation.

Abbreviation: SD, standard deviation.

^a^ Mean ± SD; *n* (%).

^b^ Wilcoxon rank sum test; Pearson's Chi‐squared test; Fisher's exact test.

**TABLE 2:** Sociodemographic characteristics, health and lifestyle factors in Bavaria stratified by sex.

Variable	*n*	Overall, *n* = 1503 (100%)^a^	Male, *n* = 756 (50%)^a^	Female, *n* = 747 (50%)^a^	*p‐Value* ^b^
BMI (in kg/m^2^)	1503	25.9 ± 0.2	26.5 ± 0.3	25.4 ± 0.3	< 0.001
BMI group^c^	1503				< 0.001
Underweight		23 (2%)	2 (0%)	21 (3%)	
Normal weight		722 (48%)	306 (40%)	417 (56%)	
Pre‐obesity		480 (32%)	297 (39%)	182 (24%)	
Obesity		278 (18%)	151 (20%)	127 (17%)	
Waist circumference (in cm)	1434	93.6 ± 0.6	99.2 ± 0.9	87.8 ± 0.8	< 0.001
Smoking	1502				0.199
Never		694 (46%)	331 (44%)	363 (49%)	
Currently		362 (24%)	204 (27%)	158 (21%)	
In the past		446 (30%)	221 (29%)	226 (30%)	
Sufficiently physically active^d^	1503				0.108
Yes		1183 (79%)	614 (81%)	569 (76%)	
No		320 (21%)	142 (19%)	178 (24%)	
Physical activity group^e^	1503				< 0.001
Sedentary		381 (25%)	156 (21%)	225 (30%)	
Low active		337 (22%)	137 (18%)	200 (27%)	
Active		323 (21%)	154 (20%)	169 (23%)	
Very active		462 (31%)	309 (41%)	153 (20%)	

*Note:* Data are weighted to represent the Bavarian population and presented as absolute (*n*) and relative frequencies (%) for categorical and as mean ± standard error for numerical variables.

Abbreviations: BMI, body mass index; SE, standard error; WHO, World Health Organization.

^a^ Mean ± SE; *n* (%).

^b^ Design‐based Wilcoxon rank‐sum test; chi‐squared test with Rao & Scott's second‐order correction.

^c^ According to the definition of the WHO (2024).

^d^ According to the European Health Interview Survey– Physical Activity Questionnaire (Finger et al. 2015).

^e^ According to Gerrior et al. (2006).

### 3.2 Diet and Eating Motives

#### 3.2.1 Vegetarian and Vegan Diets

**FIGURE 2:**
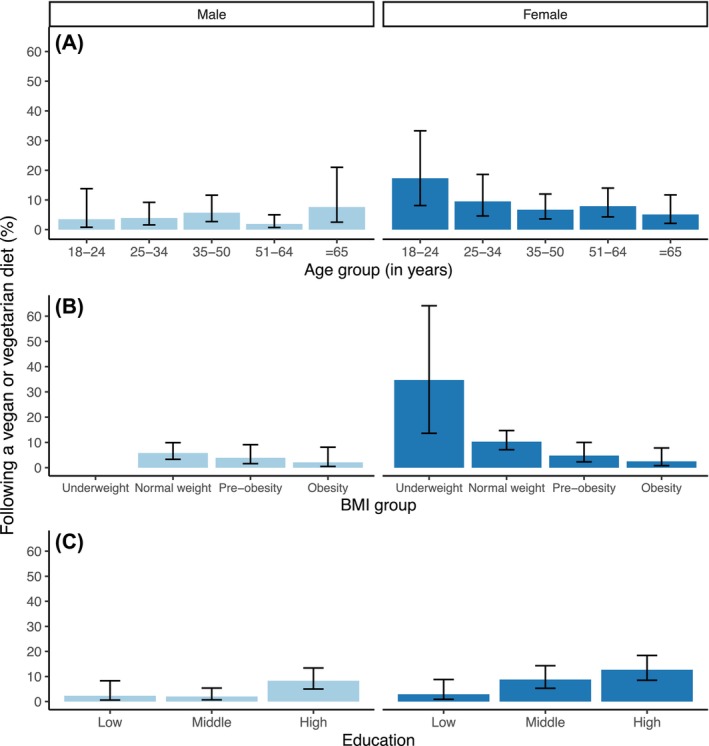
Proportion of individuals following either a vegetarian or vegan diet (pooled subsample) in Bavaria by sex and (A) age group (*n* = 1503) or (B) BMI group (*n* = 1503) or (C) education (*n* = 1502). The whiskers denote the 95% confidence interval. Data are weighted to represent the Bavarian population.

6.3% (95% CI: 4.9%, 8.1%) of the Bavarian population adhered to either a vegetarian or vegan diet, comprising 5.3% (95% CI: 4.0%, 7.0%) vegetarians and 1.0% (95% CI: 0.6%, 1.7%) vegans. Women were more likely to follow such diets (*p* = 0.016), with 8.3% (7.0% vegetarian, 1.3% vegan) compared to 4.3% of men (3.7% vegetarian, 0.6% vegan). The prevalence of vegetarian/vegan diets among females significantly decreased with age (*p‐*trend = 0.034), while no significant association with age was observed in males (*p‐*trend = 0.674) or the overall population (*p‐*trend = 0.243). Individuals following a vegetarian/vegan diet had a significantly lower average BMI of 23.6 kg/m^2^ compared to 26.1 kg/m^2^ for omnivores (*p* < 0.001). Accordingly, the proportion of vegetarians/vegans decreased with increasing BMI (*p*trend < 0.001). Education was also positively associated with the likelihood of following such diets (*p‐*trend = 0.003). Stratified by sex, the influence of education was only statistically significant in females (*p‐*trend = 0.016) and approached significance for males (*p‐*trend = 0.073) (Figure 2 and Table 3). Other types of diets practiced in Bavaria are detailed in Table S3.

**TABLE 3:** Sociodemographic characteristics, health and lifestyle factors for omnivores and individuals following either a vegetarian or vegan diet (pooled subgroup) in Bavaria.

Variable	*n*	Omnivorous, *n* = 1408 (93.7%)^b^	Vegetarian/vegan^a^, *n* = 95 (6.3%)^b^	*p‐Value* ^c^
Sex	1503			0.016
Male		95.7% (93.3%, 97.3%)	4.3% (2.7%, 6.7%)	
Female		91.7% (88.8%, 93.9%)	8.3% (6.1%, 11.2%)	
Age (in years)	1503	46.4 ± 0.7	43.5 ± 2.3	0.203
Age group	1503			0.554
18–24		90.0% (81.1%, 94.9%)	10.0% (5.1%, 18.9%)	
25–34		93.4% (88.6%, 96.3%)	6.6% (3.7%, 11.4%)	
35–50		93.8% (90.2%, 96.2%)	6.2% (3.8%, 9.8%)	
51–64		95.2% (91.9%, 97.1%)	4.8% (2.9%, 8.1%)	
≥ 65		93.7% (87.4%, 97.0%)	6.3% (3.0%, 12.6%)	
BMI (in kg/m^2^)	1503	26.1 ± 0.2	23.6 ± 0.6	< 0.001
BMI group^d^	1503			< 0.001
Underweight^e^		67.8% (39.1%, 87.3%)	32.2% (12.7%, 60.9%)	
Normal weight		91.6% (88.7%, 93.9%)	8.4% (6.1%, 11.3%)	
Pre‐obesity		95.8% (92.4%, 97.7%)	4.2% (2.3%, 7.6%)	
Obesity		97.7% (94.4%, 99.1%)	2.3% (0.9%, 5.6%)	
Education	1498			< 0.001
Low		97.4% (94.0%, 98.9%)	2.6% (1.1%, 6.0%)	
Middle		94.4% (91.2%, 96.5%)	5.6% (3.5%, 8.8%)	
High		89.5% (85.8%, 92.3%)	10.5% (7.7%, 14.2%)	
Sufficiently physically active^f^	1503			0.057
Yes		93.0% (90.8%, 94.7%)	7.0% (5.3%, 9.2%)	
No		96.3% (93.3%, 97.9%)	3.7% (2.1%, 6.7%)	
Physical activity group^g^	1503			0.149
Sedentary		95.0% (91.2%, 97.2%)	5.0% (2.8%, 8.8%)	
Low active		92.7% (88.4%, 95.5%)	7.3% (4.5%, 11.6%)	
Active		90.7% (85.5%, 94.2%)	9.3% (5.8%, 14.5%)	
Very active		95.5% (92.3%, 97.4%)	4.5% (2.6%, 7.7%)	

*Note:* Data are weighted to represent the Bavarian population and presented as relative frequencies (%) with corresponding 95% confidence interval for categorical variables. Numerical variables are depicted as mean ± standard error.

Abbreviations: BMI, body mass index; CI, confidence interval; SE, standard error; WHO, World Health Organization.

^a^ Pooled subsample of individuals self‐reporting adherence to either a vegetarian or vegan diet.

^b^ % (95% CI); Mean ± SE.

^c^ Chi‐squared test with Rao & Scott's second‐order correction; Design‐based Wilcoxon rank‐sum test.

^d^ According to the definition of the WHO (2024).

^e^ Group is very small (*n* = 23).

^f^ According to the European Health Interview Survey—Physical Activity Questionnaire (Finger et al. 2015).

^g^ According to Gerrior et al. (2006).

**FIGURE 3:**
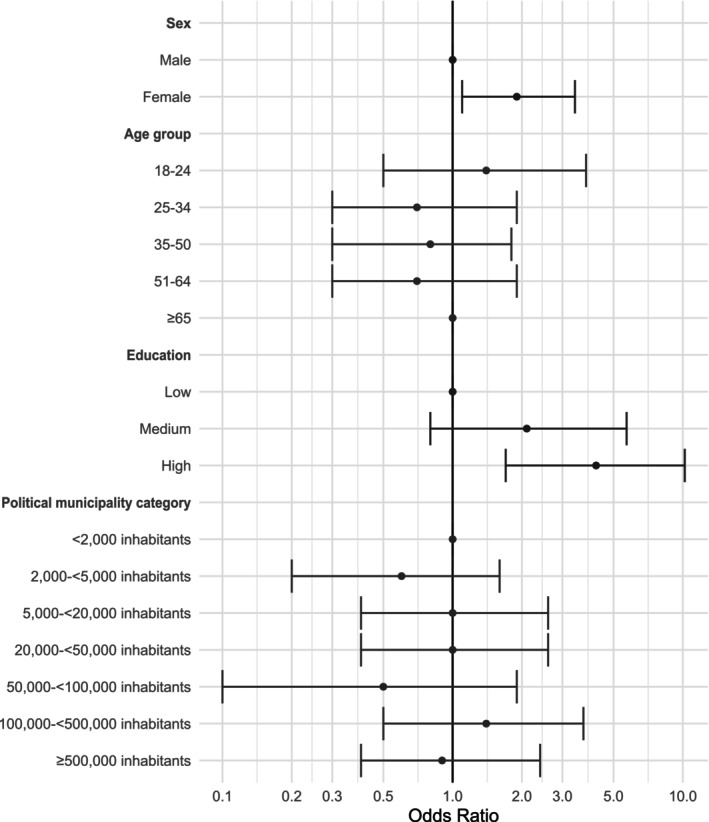
Multivariate associations between following either a vegetarian or vegan diet (pooled subsample) and the determinants sex, age group, education, political municipality category (*n* = 1502). Data are weighted to represent the Bavarian population.

The binary logistic regression analysis revealed that being female (OR: 2.3, 95% CI: 1.2–4.2, *p* = 0.033) or having a high education level (OR: 4.2, 95% CI: 1.7–10.2, *p* = 0.002) significantly increased the likelihood of following a vegetarian/vegan diet in Bavaria (Figure 3 and Table S4). In contrast, age groups and political municipality categories did not exhibit significant associations (*p* = 0.529 and *p* = 0.302, respectively).

Table 4 provides a comparative analysis of energy and nutrient intakes between omnivorous and vegetarian/vegan participants. The data were adjusted for sex and age groups. No significant differences were observed in energy intake. Protein intake was lower in the vegetarian/vegan group (62.4 g/day vs. 70.3 g/day, *p* = 0.004), including both essential (29.8 g/day vs. 35.0 g/day, *p* < 0.001) and non‐essential amino acids (32.8 g/day vs. 35.6 g/day, *p* = 0.035). Since the residuals of protein and essential amino acid intake were not normally distributed, these *p*‐values should be interpreted with caution. Total fat intake was slightly and non‐significantly lower in vegetarians/vegans, with 77.8 g/day compared to 79.3 g/day for omnivores (*p* = 0.639), with similar findings for saturated (31.9 g/day vs. 34.2 g/day, *p* = 0.157) and monounsaturated fatty acids (25.8 g/day vs. 27.1 g/day, *p* = 0.277). Polyunsaturated fatty acids, however, were marginally higher for vegetarian/vegan participants with 14.0 g/day against 12.0 g/day (*p* = 0.003), mainly attributable to higher omega‐6 fatty acids intake (12.0 g/day vs. 10.2 g/day, *p* = 0.002). Omega‐3 fatty acids were nearly identical between groups (1.9 g/day vs. 1.8 g/day, *p*‐value = 0.247). Carbohydrate intake was more than 20 g higher for vegetarians/vegans (208.0 g/day vs. 187.5 g/day, *p* = 0.007), partly due to the higher total sugar intake (88.7 g/day vs. 79.9 g/day, *p* = 0.092). Within sugars, sucrose intake showed no significant difference between groups (41.0 g/day vs. 38.4 g/day, *p* = 0.331). Fructose, however, was significantly higher in vegetarians/vegans (22.0 g/day vs. 17.4 g/day, *p* = 0.002). Fibre intake was also notably higher among vegetarians/vegans, with 23.8 g/day compared to 16.5 g/day in omnivorous individuals (*p* < 0.001).

**TABLE 4:** Daily energy and nutrient for omnivores and individuals following either a vegetarian or vegan diet (pooled subgroup) in Bavaria.

Variable	Omnivorous, *n* = 1029 (93.6%)^b^	Vegetarian/vegan^a^, *n* = 71 (6.4%)^b^	*p‐Value* ^c^
Energy intake			
Energy (in kcal)	1819.7	1840.5	0.731
Energy (in kJ)	7620.5	7706.9	0.733
Nutrient intake			
Protein (in g)^d^	70.3	62.4	0.004
Essential amino acids (in g)^d^	35.0	29.8	< 0.001
Non‐essential amino acids (in g)	35.6	32.8	0.035
Carbohydrates (in g)	187.5	208.0	0.007
Total sugar (in g)	79.9	88.7	0.092
Sucrose (in g)	38.1	41.0	0.331
Lactose (in g)^c^	7.3	5.8	0.108
Fructose (in g)^c^	17.4	22.0	0.002
Fat (in g)	79.3	77.8	0.639
Saturated fatty acid (in g)	34.2	31.9	0.157
Monounsaturated fatty acid (in g)	27.1	25.8	0.277
Polyunsaturated fatty acid (in g)	12.0	14.0	0.003
Omega‐3 fatty acid (in g)^c^	1.8	1.9	0.247
Omega‐6 fatty acid (in g)	10.2	12.0	0.002
Fibre (in g)	16.5	23.8	< 0.001
Alcohol (Ethanol) (in g)^d^, ^e^	8.4	5.7	0.042

*Note:* Data are weighted to represent the Bavarian population and presented as adjusted means. Adjustments were performed for sex and age group.

^a^ Pooled subsample of individuals self‐reporting adherence to either a vegetarian or vegan diet.

^b^ Adjusted means.

^c^ Design‐based linear regression.

^d^ Residuals were not normally distributed. *p*‐values should be interpreted with caution.

^e^ Heteroskedasticity‐robust standard error HC3 was used to account for heteroskedasticity.

The comparison of food group intakes between omnivores and those following a vegetarian/vegan diet revealed notable differences in their food intake (Table S5). The data were adjusted to a daily energy intake of 2000 kcal to ensure comparability. As expected, participants following a vegetarian/vegan diet consumed less animal‐derived products, in particular meat (6.5 g/day [median: 0.0 g/day] vs. 62.1 g/day [median: 49.9 g/day], *p* < 0.001) and meat products and sausages (5.6 g/day [median: 0.0 g/day] vs. 45.2 g/day [median: 35.1 g/day], *p* < 0.001). The consumption of fish and fish products (*p* = 0.153), butter (*p* = 0.074), milk and dairy products (*p* = 0.640) was lower in vegetarians/vegans but not statistically significant. The median egg consumption was higher for vegetarians or vegans (*p* = 0.667); however, without statistical significance (Table S5). In contrast, participants following a vegetarian/vegan diet showed a significantly higher consumption of plant‐derived foods such as vegetables (275.7 g/day [median: 233.3 g/day] vs. 179.3 g/day [median: 150.2 g/day], *p* < 0.001), legumes and leguminous vegetables (28.3 g/day [median: 10.4 g/day] vs. 12.0 g/day [median: 0.0 g/day], *p* = 0.001), and nuts, kernels and seeds (14.0 g/day [median: 5.6 g/day] vs. 6.8 g/day [median: 0.0 g/day], *p* = 0.007), but not fruits (*p* = 0.115). They also consumed more substitute products like milk and meat substitutes (65.2 g/day [median: 47.3 g/day] vs. 13.2 g/day [median: 0.0 g/day], *p* < 0.001). Beverage consumption also differed between the groups: Participants following a vegetarian/vegan diet consumed significantly more tea (469.1 g/day [median: 221.4 g/day] vs. 287.8 g/day [median: 0.0 g/day], *p* = 0.014) and significantly fewer alcoholic drinks (76.1 g/day [median: 0.0 g/day] vs. 182.8 g/day [median: 0.2 g/day], *p* = 0.018), which was also reflected in their ethanol intakes (see Table 4; 5.7 g/day vs. 8.4 g/day, *p* = 0.042). The vegetarians/vegans showed higher grain‐based staple food intake (*p* = 0.067), along with lower potato intake (*p* = 0.247), though these differences were not statistically significant.

#### 3.2.2 Eating Motives

**FIGURE 4:**
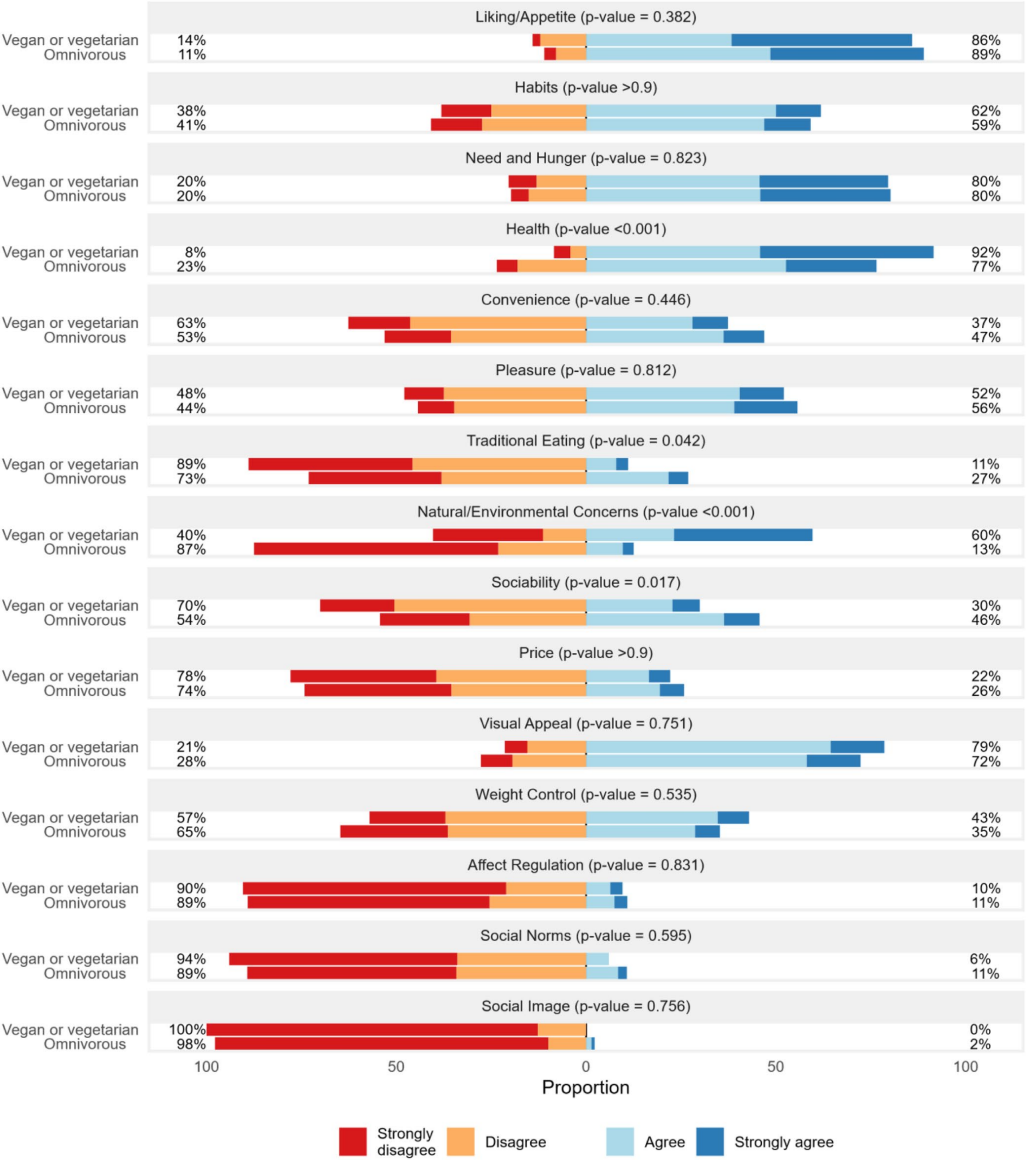
Eating motives in Bavaria by diet type (*n* = 1494). *p*‐values were computed based on chi‐squared tests with Rao & Scott's second‐order correction. Vegetarians and vegans were pooled as one subsample. Eating motives are based on Renner et al. (2012). Data are weighted to represent the Bavarian population.

Vegans and vegetarians placed significantly more importance on natural/environmental concerns (*p* < 0.001), with 60% considering them as key, compared to just 13% of omnivores. Health was also a stronger motive for those on vegetarian or vegan diets (*p* < 0.001), with 92% prioritising it versus 77% in omnivores. In contrast, traditional eating held less value for vegans and vegetarians (*p* = 0.042), with only 11% prioritising it, compared to 27% of omnivores. Sociability was similarly less important (*p* = 0.017) for vegans and vegetarians (30%) compared to omnivores (46%) (Figure 4).

A detailed description of the statistical analysis can be found in Table S6.

## 4 Discussion

The BVS III was conducted to provide comprehensive insights into dietary patterns, lifestyle and health aspects in the Bavarian population. This work specifically aimed to assess the prevalence and demographic distribution of vegetarians and vegans, alongside their motivational drivers, in comparison to the omnivorous population. Our findings reveal that 6.3% of the Bavarian population followed either a vegetarian or vegan diet. Due to the low prevalence, we pooled both groups for analysis. Women were approximately twice as likely as men to adhere to these diets. Higher education was also associated with a greater likelihood of following such diets. In contrast, neither age group nor urban or rural residence showed significant associations with adherence to a vegetarian/vegan diet. The consumption of plant‐derived foods such as vegetables, legumes, nuts and plant‐based substitutes was substantially higher in vegetarians/vegans compared to omnivores, and the diet was characterised by higher intakes of carbohydrates, fibre and polyunsaturated fatty acids, particularly omega‐6, while protein intake was moderately lower.

The prevalence of vegetarian and vegan diets in Bavaria has increased substantially over the past two decades. During the survey period (October 2021 to January 2023), 6.3% of the Bavarian population adhered to a vegetarian or vegan diet, nearly tripling the 2.2% observed in the BVS II survey conducted in 2002/2003 (Karg et al. 2004). However, the age ranges differed slightly between the studies, with BVS II including individuals aged 13–80 years (Karg et al. 2004), while BVS III focused on those aged 18–75 years. Recent comparable data for Germany or other federal states are lacking. However, annual surveys by the BMEL, involving 1000 residents aged 14 and older, showed that from 2021 to 2023, 7%–10% followed a vegetarian diet, while 1%–2% were vegans based on self‐report (BMEL 2021, 2022, 2023). Although the data are described as representative of Germany, it was denoted that a margin of error of ±3 percentage points is expected. In the unweighted data of BVS III, where selection bias is not corrected, 7.9% of participants followed a vegetarian/vegan diet. This suggests that Bavarians follow such diets either below or around the presumed national average, depending on bias correction. Surveys often have a more educated participant pool, as seen in BVS III, highlighting the need to address selection bias. Dittmann et al. (2023) identified substantial variation in the prevalence rates of vegetarians and vegans across 38 studies (with 27 conducted in Germany) between 2005 and 2022. Their analysis found percentages ranging from 1.0% to 11.2% for vegetarian diets and from 0% to 3.2% for vegan diets, which on the one hand aligns with the reported prevalences in Bavaria. On the other hand, Dittmann et al. denoted that these differences depend on the methodology and definitions applied, which remain subject to debate (Hargreaves et al. 2023). As Rosenfeld pointed out, strict definitions are challenging because individuals identifying themselves as vegetarians may occasionally consume animal‐derived foods (Rosenfeld 2018). Achieving consensus on reporting diet types is of utmost importance to ensure that findings are interpretable and comparable. However, the choice of definitions and reporting strategies is often driven by specific research questions, reflecting whether the focus might be on health outcomes, environmental impact or lifestyle and behavioural analyses. In line with BVS II (Karg et al. 2004) and the majority of studies (34 out of 38 studies) reviewed by Dittmann et al. (2023), our assessment relied on self‐reported diet types.

The proportion of vegans and vegetarians varies across Europe, and reliable data is often missing. As of July 2024, it was reported that 9% of the population in the UK followed a vegetarian or vegan diet (vegetarian: 6%; vegan: 3%) (YouGov 2024). In Austria, 11% followed either a vegetarian or vegan diet in early 2021 (Statista 2021). Austria is of particular interest due to its geographic proximity to Bavaria as well as the demographic and culinary similarities. In support of the Statista estimate, a nationwide survey among Austrian secondary school teachers (*n* = 1350) based on self‐reported diet type found 7.9% vegetarians and 2.9% vegans. Although differences in the population should be considered when interpreting comparability, the mean age of 45.8 ± 11.4 years resembled that of the BVS III sample (48.0 ± 15.1 years) (Wirnitzer et al. 2022).

The increasing prevalence of vegetarianism and veganism in Bavaria, especially among younger and higher‐educated women, reflects broader societal trends toward consciousness of personal health and environmental sustainability. However, while these findings highlight positive shifts, they reveal some challenges and complexities. The apparent sex and education disparity in the adoption of these diets was demonstrated before (Allès et al. 2017; Mensink et al. 2016). Here, women were approximately twice as likely as men to follow vegetarian or vegan diets. While health and weight control were significant eating motives for women (Figure S1), this gender gap suggests that other influences, that is, cultural and social aspects, may also play a role. A variety of studies suggested that men are less inclined to adopt vegetarianism or veganism due to traditional and stereotypical perceptions of masculinity and diet, where meat consumption is often linked to strength and virility (Bogueva et al. 2020; Camilleri et al. 2024; De Backer et al. 2020; Velzeboer et al. 2024). These circumstances were hinted at in this work, with vegans and vegetarians reporting lower priority to the eating motives ‘social norms’ and ‘social image’, compared to omnivores (albeit not statistically significant). Also, both motives were of low overall importance. Additionally, ‘traditional eating’ was significantly less influential for people who adhered to a vegetarian or vegan diet and suggestively more critical for men (not significant; Figure S1). In 2024, Camilleri et al. showed that adherence to traditional masculine norms was associated with a greater likelihood of consuming meat and a lower willingness to reduce meat consumption. Conversely, men who held non‐traditional masculine views, such as sex egalitarian beliefs, were more inclined to reduce their meat intake (Camilleri et al. 2024). Similarly, De Backer et al. (2020) demonstrated in 2020 that men who strongly identified with new forms of masculinity consumed less meat, had weaker attachments to it, were more inclined to reduce their meat intake, and held more positive attitudes toward vegetarianism.

A high education level significantly increased the likelihood of following a vegetarian or vegan diet in Bavaria, while the political municipality category had no effect. The political municipality category was included in the analysis to assess whether regional characteristics, that is, rurality or urbanity, might influence the likelihood of following these diets. In DEGS1 (conducted from 2008 to 2011), community size increased the odds to follow a vegetarian diet (Mensink et al. 2016). However, the findings here suggest that education played a far more decisive role in Bavaria. The distinct influence of education on adopting a vegetarian/vegan diet underscores the importance of knowledge and awareness in shaping dietary choices. Those with higher education levels are possibly more likely to be aware of the benefits of vegetarianism or veganism. Recent findings emphasise the broader educational deficits that possibly limit informed dietary decision‐making, even among healthcare professionals. A survey of registered dietitians in the UK and Ireland revealed widespread misconceptions and insufficient education on plant‐based nutrition: 79% felt they had not received sufficient education on whole food plant‐based diets and 75% held misconceptions about plant‐derived protein quality (Metoudi et al. 2025). Similar but more widespread knowledge gaps are apparent among physicians. Of 248 US physicians, only 13.5% said they were confident in advising patients on nutrition, despite overwhelmingly recognising its importance (78.4%) in clinical care (Harkin et al. 2019). Van Horn et al. (2019) reported that medical education in the US continues to lack standardised medical nutrition education and that there is a need for competency‐based nutrition programmes. A recent perspective called for a systemic upgrade of medical curricula to incorporate evidence‐based plant‐based nutrition, not only to prevent non‐communicable diseases but also to reduce the risk of future pandemics linked to dietary and environmental factors (Gatterer et al. 2022). Limited access to nutrition education may exacerbate existing health inequities, as less‐educated individuals may be less aware of the health benefits and practical implementation of plant‐based diets. While higher education is generally associated with higher income (Card 1999), the key barrier in this context appears to be educational rather than financial. Although plant‐based foods are often perceived as more expensive, evidence from a global modelling study shows that healthy and sustainable dietary patterns are already 22%–34% less expensive than current diets in upper‐middle to high‐income countries, with cost savings expected to increase further by the year 2050. Especially forms of vegetarian and vegan diets were generally most affordable (Springmann et al. 2021). This finding suggests that plant‐based diets do not constitute a financial overburden. This was factually supported by several national studies and subgroup analyses demonstrating greater affordability of vegetarian and vegan diets (Hohoff et al. 2022; Kahleova et al. 2023; Pais et al. 2022).

In this study, participants following a vegetarian/vegan diet consumed higher quantities of substitute products and plant‐based protein sources like legumes, nuts and seeds, but also lower amounts of (protein‐rich) foods of animal origin. This resulted in a lower protein intake (including essential amino acids) of 62.4 g/day compared to 70.3 g/day for omnivores. However, lower intake does not necessarily imply inadequate supply. Based on the EFSA recommendation of 0.83 g protein per day and kg body weight (considering the 97.5th percentile of the distribution of the requirement and utilisation efficacy of dietary protein) (EFSA Panel on Dietetic Products and Allergies 2012), the adjusted mean protein intake of 62.4 g per day in the vegetarian/vegan group would be sufficient for a person with a body weight of up to 75 kg. The intake also fell within the recommended range by the DGE (2017) of48 to 677 g/day for adults based on normal weight. Omnivores exceeded this reference with 70.3 g/day. While the absolute intake of essential amino acids was lower for vegetarians/vegans, their proportion relative to total protein was similar (approx. 50%) in both groups, indicating qualitatively similar amino acid profiles. A study using the EPIC‐Oxford cohort (Schmidt et al. 2016) showed that although the intake of most essential amino acids was significantly lower in male vegetarians and vegans compared to meat‐eaters, blood plasma concentrations did not fully mirror these differences. Plasma concentrations were only marginally lower in vegans and not reduced in vegetarians. Intakes and plasma concentrations of amino acids were not strongly correlated (Schmidt et al. 2016), suggesting that lower intake does not necessarily translate to lower availability. The German NuEva study also reported lower protein intakes in vegetarians and vegans compared to omnivores. In NuEva, median intakes in both groups were below the reference values set by the DGE (Dawczynski et al. 2022), which was, as reported above, not the case in Bavaria. The study emphasised that well‐planned vegetarian diets can be nutritionally adequate and beneficial for the prevention and management of non‐communicable diseases (Dawczynski et al. 2022) in support of the position of the AND (Dawczynski et al. 2022; Melina et al. 2016).

In 2019, the EAT‐Lancet Commission provided plant‐forward dietary recommendations aiming at incorporating all aspects of sustainable nutrition, that is, nutritionally beneficial with low environmental, fiscal and social burdens (Willett et al. 2019). The dietary patterns observed among vegetarians/vegans in Bavaria, characterised by, beyond others, lower saturated fat and higher fibre intake compared to omnivores, align well with these principles. Lower saturated fat intake supports cardiovascular health and reduces environmental impact, as animal‐sourced fats are generally associated with a higher saturated fat content and ecological footprint (Mertens et al. 2019; Willett et al. 2019). The significantly higher fibre intake promotes health and disease prevention (Ramezani et al. 2024) and is indicative of minimally processed plant‐derived foods, which are also emphasised in the FAO's sustainability framework (Burlingame and Dernini 2012). These dietary characteristics have been associated with favourable health outcomes in large cohort studies based on AHS‐2 and EPIC‐Oxford, including reduced risks of type 2 diabetes, hypertension and certain cancers (Key et al. 2022; Orlich and Fraser 2014). Our own recently published analysis based on BVS III data further supports ecological alignment. Compared to omnivores, the combined group of vegetarians and vegans had 24% lower greenhouse gas emissions and 26% lower land use per 2500 kcal, while the water footprint was comparable (Gimpfl et al. 2025). These results underscore the environmental advantages of vegetarian/vegan diets and provide regionally specific evidence supporting the EAT‐Lancet concept of the Planetary Health Diet, emphasising their potential as health‐promoting and environmentally sustainable dietary strategies. However, the Planetary Health Diet has been criticised for potentially falling short in meeting certain micronutrient requirements, particularly vitamin B12, calcium, iron and zinc, which are typically more abundant and bioavailable in animal‐derived foods (Beal et al. 2023). In addition, concerns have been raised about the affordability and accessibility of the diet, especially in low‐income settings (Hirvonen et al. 2020), highlighting the need for careful adaptation and implementation strategies to ensure nutritional adequacy and equity across different populations. To address these and other shortfalls, the *Lancet* announced a scientific update, the 2025 EAT‐*Lancet* Commission, to take place on the 3rd of October 2025 (The EAT‐Lancet Commission 2025).

The BVS III was conducted under challenging conditions during the COVID‐19 pandemic, which affected participant recruitment and data collection logistics. Despite these difficulties, the study maintained methodological rigour through strategic oversampling and the application of weighting factors to correct for demographic imbalances, ensuring representativeness of the Bavarian adult population. Diet type classification in this study was based on self‐reported dietary categories. While this approach was deliberately chosen to ensure methodological consistency with the preceding BVS II, it is subject to known limitations. Dietary self‐identification can be influenced by personal interpretation, recall bias or social desirability, and occasional meat consumption was not explicitly queried or corrected for. Additionally, the low but unintuitive consumption of meat and meat products/sausages observed in the vegan/vegetarian group was also likely due to methodological limitations in the dietary assessment tool. The GloboDiet software and the underlying BLS database use predefined composite recipes, which occasionally include small amounts of animal‐based ingredients, even in recipes generally considered plant‐based, for example, the use of bacon, ham or meat broths in risotto, stews and soups.

## 5 Conclusions

The prevalences of vegetarianism and veganism have increased in Bavaria over the past two decades, reflecting global trends toward health consciousness and environmental sustainability. While being female and higher education emerged as strong predictors, rurality and age had no significant effect, suggesting that vegetarianism and veganism are not limited to urban settings but are becoming more common across Bavaria, including rural areas. Health and environmental concerns were important eating motives among vegetarians/vegans, whereas traditional eating and sociability were less influential than among omnivores. This shift could pose challenges, such as social isolation, especially in cultures where communal eating and traditional foods are central to social identity. Addressing such aspects will be important to support long‐term adherence to vegetarian, vegan and more broadly plant‐forward diets. In addition, meat consumption has declined compared to BVS II, a trend also reflected in the German food balance sheets. This decline may be more impactful than the rise in strict plant‐based diets. The decreasing intake of meat and sausage products indicates a gradual dietary change rather than an absolute dietary overhaul. While environmental concerns were subsidiary eating motives, health was overall important. Linking individual health goals with planetary health will be essential to reinforce the already observed gradual change in Bavaria. Reinforcing existing tendencies may pose a more inclusive and socially sustainable approach to public health and environmental goals. As dietary choices continue to evolve, integrating environmental, social and health with current dietary developments will be essential in shaping future nutritional guidelines and public health initiatives.

## Author Contributions

S.G.: data curation, formal analysis, methodology, writing – original draft, writing – review and editing. F.R.: data curation, writing – review and editing. N.W.: data curation, writing – review and editing. N.O.: writing – review and editing. C.R.: funding acquisition, project administration, writing – review and editing. M.S.: project administration, writing – review and editing. M.K.: project administration, writing – review and editing. J.L.: conceptualisation, funding acquisition, resources, writing – review and editing. K.G.: conceptualisation, funding acquisition, methodology, resources, supervision, writing – review and editing. S.G. drafted the manuscript. All authors (S.G., F.R., N.W., N.O., C.R., M.S., M.K., J.L., K.G.) made significant contributions to the conception or design of the study, as well as to the acquisition, analysis or interpretation of data. Each author reviewed the manuscript critically, provided final approval, and agreed to be accountable for all aspects of the work.

## Ethics Statement

The survey protocol was reviewed and approved by the ethics review board at the Medical Faculty of the Ludwig‐Maximilians‐University of Munich, approval number 20‐0334. The investigation adhered to the principles of the Declaration of Helsinki, and written informed consent was obtained from all participants prior to their involvement in the survey.

## Conflicts of Interest

The authors declare no conflicts of interest.

## Supporting information


**Table S1:** Description of the BVS III study sample with at least two 24‐h dietary recalls (excluding underreporters) stratified by sex. Data are presented as absolute (*n*) and relative frequencies (%) for categorical variables. Age is displayed as mean ± SD.
**Table S2:** Demographic characteristics in Bavaria stratified by sex and based on the BVS III study sample with at least two 24‐h dietary recalls (excluding underreporters). Data are weighted to represent the Bavarian population and presented as absolute (*n*) and relative frequencies (%) for categorical and as mean ± SE for numerical variables.
**Table S3:** Diet types in Bavaria. Multiple answers were possible. Data are weighted to represent the Bavarian population and presented as absolute (*n*) and relative frequencies (%).
**Table S4:** Multivariate associations between following vegetarian or vegan diet (collectively pooled as one subgroup) and the determinants sex, age group, education, political municipality category (*n* = 1502). Data are weighted to represent the Bavarian population. Associations are presented as OR with 95% CI.
**Table S5:** Food group consumption patterns for omnivores and individuals following either a vegetarian or vegan diet (pooled subgroup) in Bavaria. Intakes of food and beverage groups are in g/2000 kcal. Bold descriptions represent the superordinate food groups. Data are weighted to represent the Bavarian population and presented as mean ± SE with the median in parenthesis.
**Table S6:** Test statistics to the eating motives analyses (*n* = 1494). Vegetarians and vegans were pooled as one subsample. Data are weighted to represent the Bavarian population.
**Figure S1:** Eating motives in Bavaria by sex (*n* = 1494). *p*‐Values were computed based on chi‐squared tests with Rao & Scott's second‐order correction. Eating motives are based on Renner et al. (2012). Data are weighted to represent the Bavarian population.

## Data Availability

The data that supports the findings of this study will be available following the publication of the primary results in the coming months. Afterward, they can be requested upon reasonable justification, including a description of the intended analysis and approval from the project partners. All requests must ensure proper use, with any misuse strictly prohibited.
