# Supplementary material for: Self‐Reported Adherence to Vegetarian and Vegan Diets: Insights From the 3rd Bavarian Food Consumption Survey
Source: Nutr Bull. 2025 Sep 9;50(4):617–32. doi: 10.1111/nbu.70029 (PMC12621161; doi:10.1111/nbu.70029)
Supplement: Supplementary file 1 — Table S1: Description of the BVS III study sample with at least two 24‐h dietary recalls (excluding underreporters) stratified by sex. Data are presented as absolute (n) and relative frequencies (%) for categorical variables. Age is displayed as mean ± SD. Table S2: Demographic characteristics in Bavaria stratified by sex and based on the BVS III study sample with at least two 24‐h dietary recalls (excluding underreporters). Data are weighted to represent the Bavarian population and presented as absolute (n) and relative frequencies (%) for categorical and as mean ± SE for numerical variables. Table S3: Diet types in Bavaria. Multiple answers were possible. Data are weighted to represent the Bavarian population and presented as absolute (n) and relative frequencies (%). Table S4: Multivariate associations between following vegetarian or vegan diet (collectively pooled as one subgroup) and the determinants sex, age group, education, political municipality category (n = 1502). Data are weighted to represent the Bavarian population. Associations are presented as OR with 95% CI. Table S5: Food group consumption patterns for omnivores and individuals following either a vegetarian or vegan diet (pooled subgroup) in Bavaria. Intakes of food and beverage groups are in g/2000 kcal. Bold descriptions represent the superordinate food groups. Data are weighted to represent the Bavarian population and presented as mean ± SE with the median in parenthesis. Table S6: Test statistics to the eating motives analyses (n = 1494). Vegetarians and vegans were pooled as one subsample. Data are weighted to represent the Bavarian population. Figure S1: Eating motives in Bavaria by sex (n = 1494). p‐Values were computed based on chi‐squared tests with Rao & Scott's second‐order correction. Eating motives are based on Renner et al. (2012). Data are weighted to represent the Bavarian population. [file NBU-50-617-s001.docx]

Supplementary Material

# Supplementary Tables

Supplementary Table 1 Description of the BVS III study sample with at least two 24-h dietary recalls (excluding underreporters) stratified by sex. Data are presented as absolute (n) and relative frequencies (%) for categorical variables. Age is displayed as mean ± SD.

| **Variable** | **n** | **Overall**, n = 1100 (100%)*^1^* | **Male**, n = 485 (44%)*^1^* | **Female**, n = 615 (56%)*^1^* | **p-value***^2^* |
| --- | --- | --- | --- | --- | --- |
| **Age (in years)** | 1,100 | 49.3 ± 14.9 | 49.3 ± 14.8 | 49.3 ± 14.9 | >0.9 |
| **Age group (in years)** | 1,100 |  |  |  | >0.9 |
| 18-24 |  | 71 (6%) | 30 (6%) | 41 (7%) |  |
| 25-34 |  | 156 (14%) | 71 (15%) | 85 (14%) |  |
| 35-50 |  | 297 (27%) | 126 (26%) | 171 (28%) |  |
| 51-64 |  | 387 (35%) | 176 (36%) | 211 (34%) |  |
| ≥65 |  | 189 (17%) | 82 (17%) | 107 (17%) |  |
| **Education** | 1,100 |  |  |  | 0.010 |
| Low |  | 220 (20%) | 98 (20%) | 122 (20%) |  |
| Middle |  | 317 (29%) | 118 (24%) | 199 (32%) |  |
| High |  | 563 (51%) | 269 (55%) | 294 (48%) |  |
| **Civil status** | 1,099 |  |  |  | <0.001 |
| Single |  | 148 (13%) | 72 (15%) | 76 (12%) |  |
| Unmarried - in a partnership |  | 146 (13%) | 65 (13%) | 81 (13%) |  |
| Married |  | 697 (63%) | 321 (66%) | 376 (61%) |  |
| Widowed |  | 29 (3%) | 4 (1%) | 25 (4%) |  |
| Divorced |  | 79 (7%) | 23 (5%) | 56 (9%) |  |
| **Living situation** | 1,100 |  |  |  | 0.371 |
| Living alone in a private household |  | 171 (16%) | 67 (14%) | 104 (17%) |  |
| Living in a private household with family/friends or other persons |  | 926 (84%) | 417 (86%) | 509 (83%) |  |
| Community-oriented living arrangement |  | 1 (0%) | 0 (0%) | 1 (0%) |  |
| Other |  | 2 (0%) | 1 (0%) | 1 (0%) |  |
| **Employment** | 1,100 |  |  |  | 0.002 |
| Employed |  | 705 (64%) | 326 (67%) | 379 (62%) |  |
| Marginally, occasionally or irregularly employed |  | 29 (3%) | 4 (1%) | 25 (4%) |  |
| In vocational training/apprenticeship/retraining |  | 18 (2%) | 11 (2%) | 7 (1%) |  |
| Currently not employed: unemployed or job-seeking, on parental leave |  | 59 (5%) | 18 (4%) | 41 (7%) |  |
| Retired, pensioner, homemaker |  | 246 (22%) | 105 (22%) | 141 (23%) |  |
| Other (e.g., pupil, student, assisting family member) |  | 43 (4%) | 21 (4%) | 22 (4%) |  |
| Abbreviations: SD standard deviation | | | | | |
| *^1^*Mean ± SD; n (%) | | | | | |
| *^2^*Wilcoxon rank sum test; Pearson's Chi-squared test; Fisher's exact test | | | | | |

Supplementary Table 2 Demographic characteristics in Bavaria stratified by sex and based on the BVS III study sample with at least two 24-h dietary recalls (excluding underreporters). Data are weighted to represent the Bavarian population and presented as absolute (n) and relative frequencies (%) for categorical and as mean ± SE for numerical variables.

| **Variable** | **n** | **Overall**, n = 1100 (100%)*^1^* | **Male**, n = 558 (51%)*^1^* | **Female**, n = 542 (49%)*^1^* | **p-value***^2^* |
| --- | --- | --- | --- | --- | --- |
| **BMI (in kg/m^2^)** | 1,100 | 26.0 ± 0.3 | 26.8 ± 0.3 | 25.3 ± 0.4 | <0.001 |
| **BMI group***^3^* | 1,100 |  |  |  | <0.001 |
| Underweight |  | 18 (2%) | 1 (0%) | 17 (3%) |  |
| Normal weight |  | 514 (47%) | 201 (36%) | 313 (58%) |  |
| Pre-obesity |  | 362 (33%) | 244 (44%) | 118 (22%) |  |
| Obesity |  | 206 (19%) | 112 (20%) | 94 (17%) |  |
| **Waist circumference (in cm)** | 1,061 | 93.9 ± 0.8 | 99.8 ± 1.0 | 87.5 ± 1.1 | <0.001 |
| **Smoking** | 1,099 |  |  |  | 0.301 |
| Never |  | 554 (50%) | 271 (49%) | 283 (52%) |  |
| Currently |  | 184 (17%) | 109 (20%) | 75 (14%) |  |
| In the past |  | 362 (33%) | 177 (32%) | 184 (34%) |  |
| **Sufficiently physically active***^4^* | 1,100 |  |  |  | 0.147 |
| Yes |  | 864 (79%) | 454 (81%) | 410 (76%) |  |
| No |  | 236 (21%) | 104 (19%) | 132 (24%) |  |
| **Physical activity group*^5^*** | 1,100 |  |  |  | <0.001 |
| Sedentary |  | 258 (23%) | 97 (17%) | 162 (30%) |  |
| Low active |  | 261 (24%) | 101 (18%) | 160 (29%) |  |
| Active |  | 256 (23%) | 133 (24%) | 122 (23%) |  |
| Very active |  | 326 (30%) | 227 (41%) | 98 (18%) |  |
| Abbreviations: BMI body mass index, SE standard error, WHO World Health Organization | | | | | |
| *^1^*Mean ± SE; n (%) | | | | | |
| *^2^*Design-based Wilcoxon rank-sum test; Chi-squared test with Rao & Scott's second-order correction | | | | | |
| *^3^*According to the definition of the WHO (2024) | | | | | |
| *^4^*According to the European Health Interview Survey - Physical Activity Questionnaire (Finger et al. 2015) | | | | | |
| *^5^*According to Gerrior et al. (2006) | | | | | |

Supplementary Table 3 Diet types in Bavaria. Multiple answers were possible. Data are weighted to represent the Bavarian population and presented as as absolute (n) and relative frequencies (%).

| **Diet Types** | **Overall**, n = 1,503 (100%)*^1^* | **Male**, n = 756 (50%)*^1^* | **Female**, n = 747 (50%)*^1^* | **p-value***^2^* |
| --- | --- | --- | --- | --- |
| **No specific diet type** | 1,343 (89.3%) | 696 (92.0%) | 647 (86.6%) | 0.018 |
| **Vegetarian or vegan** | 95 (6.3%) | 33 (4.3%) | 62 (8.3%) | 0.016 |
| Vegetarian | 80 (5.3%) | 28 (3.7%) | 52 (7.0%) | 0.038 |
| Vegan | 15 (1.0%) | 5 (0.6%) | 10 (1.3%) | 0.156 |
| **Other diet types*^3^*** | 67 (4.5%) | 28 (3.7%) | 39 (5.2%) | 0.354 |
| Fasting | 30 (2.0%) | 12 (1.6%) | 18 (2.4%) | 0.568 |
| Diets reduced in or free of sugar or carbohydrates | 14 (0.9%) | 4 (0.6%) | 10 (1.3%) | 0.171 |
| Other | 24 (1.6%) | 12 (1.6%) | 12 (1.6%) | >0.9 |
| *^1^*n (%) | | | | |
| *^2^*Chi-squared test with Rao & Scott's second-order correction | | | | |
| *^3^*Categorization of open text answers | | | | |

Supplementary Table 4 Multivariate associations between following vegetarian or vegan diet (collectively pooled as one subgroup) and the determinants sex, age group, education, political municipality category (n = 1,502). Data are weighted to represent the Bavarian population. Associations are presented as OR with 95% CI.

|  | **OR (95% CI)** | **p-value** |
| --- | --- | --- |
| **Sex** |  |  |
| Male | *Reference* |  |
| Female | 2.3 (1.2, 4.2) | 0.033 |
| **Age group (in years)** |  |  |
| 18 – 24 years | 1.4 (0.5, 3.8) | 0.531 |
| 25 – 34 years | 0.7 (0.3, 1.9) | 0.529 |
| 35 – 50 years | 0.8 (0.3, 1.8) | 0.547 |
| 51 – 64 years | 0.7 (0.3, 1.9) | 0.533 |
| ≥65 | *Reference* |  |
| **Education** |  |  |
| Low | *Reference* |  |
| Middle | 2.1 (0.8, 5.7) | 0.133 |
| High | 4.2 (1.7, 10.2) | 0.002 |
| **Political municipality category** |  |  |
| <2,000 inhabitants | *Reference* |  |
| 2,000-<5,000 inhabitants | 0.6 (0.2, 1.6) | 0.313 |
| 5,000-<20,000 inhabitants | 1.0 (0.4, 2.6) | >0.9 |
| 20,000-<50,000 inhabitants | 1.0 (0.4, 2.6) | >0.9 |
| 50,000-<100,000 inhabitants | 0.5 (0.1, 1.9) | 0.301 |
| 100,000-<500,000 inhabitants | 1.4 (0.5, 3.7) | 0.478 |
| ≥500,000 inhabitants | 0.9 (0.4, 2.4) | >0.9 |
| Abbreviations: CI confidence interval, OR odds ratio | | |

Supplementary Table 5 Food group consumption patterns for omnivores and individuals following either a vegetarian or vegan diet (pooled subgroup) in Bavaria. Intakes of food and beverage groups are in g/2,000 kcal. Bold descriptions represent the superordinate food groups. Data are weighted to represent the Bavarian population and presented as mean ± SE with the median in parenthesis.

| **Food group (in g)** | **Overall**, n = 1100 (100%)*^2^* | **Omnivorous**, n = 1029 (93.6%)*^2^* | **Vegan/vegetarian***^1^*, n = 71(6.4%)*^2^* | **p-value***^3^* |
| --- | --- | --- | --- | --- |
| **Meat** | 58.6 ± 3.0 (47.2) | 62.1 ± 3.2 (49.9) | 6.5 ± 2.3 (0.0) | <0.001 |
| Meat, uncategorized | 7.3 ± 1.0 (0.0) | 7.8 ± 1.0 (0.0) | 1.2 ± 0.8 (0.0) | <0.001 |
| Beef | 11.5 ± 1.3 (0.0) | 12.2 ± 1.4 (0.0) | 1.7 ± 1.3 (0.0) | <0.001 |
| Veal | 0.9 ± 0.3 (0.0) | 0.9 ± 0.3 (0.0) | 0.0 ± 0.0 (0.0) | 0.002 |
| Pork | 16.3 ± 1.5 (0.0) | 17.3 ± 1.6 (0.0) | 1.6 ± 1.0 (0.0) | <0.001 |
| Mutton/Lamb | 0.4 ± 0.2 (0.0) | 0.5 ± 0.2 (0.0) | 0.0 ± 0.0 (0.0) | 0.024 |
| Horse, goat, rabbit meat, and game mammals | 0.5 ± 0.3 (0.0) | 0.6 ± 0.3 (0.0) | 0.0 ± 0.0 (0.0) | 0.015 |
| Poultry and game poultry | 21.3 ± 2.2 (0.0) | 22.6 ± 2.3 (0.0) | 1.9 ± 1.7 (0.0) | <0.001 |
| Variety meat and offal | 0.3 ± 0.2 (0.0) | 0.3 ± 0.2 (0.0) | 0.0 ± 0.0 (0.0) | 0.108 |
| **Meat and sausage products** | 42.7 ± 2.2 (31.3) | 45.2 ± 2.3 (35.1) | 5.6 ± 2.0 (0.0) | <0.001 |
| Sausage and sausage products | 34.6 ± 2.1 (20.5) | 36.7 ± 2.2 (23.1) | 3.9 ± 1.8 (0.0) | <0.001 |
| Ham and cured meat | 7.9 ± 0.7 (0.0) | 8.3 ± 0.8 (0.0) | 1.7 ± 0.7 (0.0) | <0.001 |
| Canned meat | 0.2 ± 0.2 (0.0) | 0.2 ± 0.2 (0.0) | 0.0 ± 0.0 (0.0) | 0.199 |
| **Fish and fish products** | 19.1 ± 1.7 (0.0) | 19.6 ± 1.8 (0.0) | 10.9 ± 3.7 (0.0) | 0.153 |
| Fish, fresh and frozen | 10.2 ± 1.2 (0.0) | 10.3 ± 1.2 (0.0) | 8.3 ± 3.6 (0.0) | 0.907 |
| Canned fish | 7.8 ± 1.1 (0.0) | 8.2 ± 1.2 (0.0) | 1.5 ± 0.7 (0.0) | <0.001 |
| Other fish products | 1.1 ± 0.3 (0.0) | 1.1 ± 0.4 (0.0) | 1.2 ± 0.8 (0.0) | 0.383 |
| **Eggs** | 20.5 ± 1.5 (10.1) | 20.5 ± 1.6 (9.1) | 19.9 ± 3.1 (17.5) | 0.667 |
| **Milk and dairy products** | 173.8 ± 6.4 (144.5) | 174.0 ± 6.8 (149.1) | 169.9 ± 19.5 (128.5) | 0.640 |
| Milk | 72.5 ± 4.9 (29.9) | 73.4 ± 5.1 (29.7) | 60.2 ± 10.7 (42.0) | 0.846 |
| Cream | 1.6 ± 0.2 (0.0) | 1.5 ± 0.2 (0.0) | 3.0 ± 1.8 (0.0) | 0.219 |
| Cream cheese, quark | 14.6 ± 2.0 (0.0) | 14.5 ± 2.1 (0.0) | 16.7 ± 5.0 (0.0) | 0.282 |
| Fermented dairy products | 41.8 ± 3.3 (0.0) | 41.8 ± 3.4 (0.0) | 41.7 ± 13.3 (0.0) | 0.232 |
| Other milk-based and dairy products | 6.0 ± 1.7 (0.0) | 6.3 ± 1.8 (0.0) | 2.1 ± 1.4 (0.0) | 0.075 |
| Cheese | 37.2 ± 1.6 (28.7) | 36.6 ± 1.7 (28.7) | 46.1 ± 5.5 (44.7) | 0.084 |
| **Butter** | 9.4 ± 0.5 (6.6) | 9.6 ± 0.5 (6.8) | 7.1 ± 1.7 (3.0) | 0.074 |
| **Cooking oils and fats (excluding butter)** | 11.6 ± 0.6 (8.5) | 11.3 ± 0.6 (8.2) | 15.8 ± 1.6 (14.8) | 0.005 |
| Margarine | 1.4 ± 0.2 (0.0) | 1.4 ± 0.2 (0.0) | 1.7 ± 0.6 (0.0) | 0.899 |
| Plant-based fats and oils | 5.7 ± 0.3 (3.8) | 5.4 ± 0.3 (3.6) | 9.8 ± 1.2 (8.4) | 0.001 |
| Mayonnaise and other fat-based products | 3.6 ± 0.4 (0.0) | 3.6 ± 0.4 (0.0) | 2.7 ± 1.0 (0.0) | 0.229 |
| Animal-based fats and oils | 0.1 ± 0.0 (0.0) | 0.1 ± 0.0 (0.0) | 0.0 ± 0.0 (0.0) | <0.001 |
| Cooking fats and oils, uncategorized | 0.9 ± 0.1 (0.0) | 0.8 ± 0.1 (0.0) | 1.5 ± 0.7 (0.0) | 0.557 |
| **Bread and bakery products** | 125.7 ± 3.2 (121.2) | 124.4 ± 3.2 (120.6) | 144.4 ± 16.1 (130.9) | 0.452 |
| White bread, crispbread, bread rolls | 49.3 ± 2.3 (40.5) | 49.4 ± 2.5 (41.4) | 46.8 ± 6.6 (28.7) | 0.728 |
| Other bread | 26.0 ± 1.4 (17.3) | 25.7 ± 1.4 (17.5) | 29.8 ± 6.0 (12.0) | 0.855 |
| Baked goods and pastries | 50.4 ± 2.7 (37.0) | 49.2 ± 2.5 (34.5) | 67.7 ± 18.3 (48.1) | 0.460 |
| **Grain-based staple foods** | 108.5 ± 5.0 (81.8) | 106.8 ± 5.3 (80.4) | 134.0 ± 15.4 (112.6) | 0.067 |
| Flour | 4.5 ± 1.0 (0.0) | 4.4 ± 1.0 (0.0) | 6.8 ± 2.2 (0.2) | 0.206 |
| Rice | 24.1 ± 3.1 (0.0) | 23.9 ± 3.3 (0.0) | 26.9 ± 7.0 (0.0) | 0.421 |
| Grains (excluding rice) | 10.2 ± 1.4 (0.0) | 9.5 ± 1.4 (0.0) | 20.5 ± 6.4 (6.1) | 0.002 |
| Other grain products | 4.6 ± 0.5 (0.0) | 4.5 ± 0.5 (0.0) | 6.4 ± 2.1 (0.0) | 0.468 |
| **Pasta products** | 65.1 ± 4.2 (42.3) | 64.5 ± 4.4 (41.0) | 73.3 ± 14.0 (54.0) | 0.743 |
| **Wholegrain products** | 18.9 ± 1.2 (6.3) | 18.7 ± 1.2 (6.3) | 20.6 ± 4.2 (8.4) | 0.807 |
| Wholegrain pasta products | 1.0 ± 0.5 (0.0) | 1.0 ± 0.5 (0.0) | 2.1 ± 1.9 (0.0) | 0.435 |
| Muesli | 3.4 ± 0.5 (0.0) | 3.3 ± 0.5 (0.0) | 5.2 ± 1.9 (0.0) | 0.192 |
| Wholegrain bread and bread rolls | 14.4 ± 1.0 (0.0) | 14.5 ± 1.1 (0.0) | 13.3 ± 3.5 (0.0) | 0.387 |
| **Potatoes and potato products** | 71.6 ± 3.7 (53.0) | 72.3 ± 3.9 (54.9) | 61.2 ± 10.8 (30.3) | 0.247 |
| Potatoes, fresh | 66.7 ± 3.6 (49.0) | 67.3 ± 3.8 (49.8) | 57.9 ± 10.3 (30.3) | 0.350 |
| Potato products | 4.8 ± 1.2 (0.0) | 5.0 ± 1.3 (0.0) | 3.3 ± 1.5 (0.0) | 0.395 |
| **Vegetables** | 185.5 ± 6.2 (154.4) | 179.3 ± 6.4 (150.2) | 275.7 ± 22.1 (233.3) | <0.001 |
| Vegetables, uncategorized | 15.0 ± 1.5 (0.0) | 14.7 ± 1.5 (0.0) | 18.1 ± 6.4 (0.0) | 0.768 |
| Salad vegetables | 20.9 ± 1.4 (10.8) | 20.7 ± 1.4 (10.6) | 24.2 ± 4.8 (14.9) | 0.772 |
| Leafy and stalk vegetables | 4.8 ± 1.0 (0.0) | 4.6 ± 1.0 (0.0) | 7.9 ± 2.8 (0.0) | 0.342 |
| Cabbage vegetables | 17.7 ± 1.6 (0.0) | 17.0 ± 1.7 (0.0) | 26.9 ± 6.6 (11.7) | 0.074 |
| Sprout and leek vegetables | 18.5 ± 1.2 (10.7) | 17.8 ± 1.2 (10.4) | 29.6 ± 5.9 (18.9) | 0.002 |
| Fruit vegetables | 84.3 ± 4.1 (58.0) | 81.1 ± 4.2 (55.2) | 130.2 ± 17.3 (114.1) | 0.002 |
| Root and tuber vegetables | 16.8 ± 1.6 (4.1) | 16.2 ± 1.7 (3.9) | 25.2 ± 4.0 (15.6) | 0.011 |
| Oil fruits | 0.9 ± 0.2 (0.0) | 0.9 ± 0.2 (0.0) | 0.6 ± 0.3 (0.0) | 0.662 |
| Mushrooms | 3.6 ± 0.6 (0.0) | 3.5 ± 0.6 (0.0) | 5.0 ± 2.0 (0.0) | 0.597 |
| Vegetable products | 3.0 ± 0.6 (0.0) | 2.7 ± 0.6 (0.0) | 7.9 ± 3.1 (0.0) | 0.068 |
| **Legumes and pulses** | 13.1 ± 2.0 (0.0) | 12.0 ± 2.1 (0.0) | 28.3 ± 5.8 (10.4) | 0.001 |
| **Fruits** | 130.4 ± 7.3 (95.9) | 128.4 ± 7.7 (93.9) | 158.4 ± 20.3 (145.8) | 0.115 |
| Fruits, uncategorized | 10.6 ± 2.3 (0.0) | 10.2 ± 2.4 (0.0) | 15.9 ± 8.2 (0.0) | 0.719 |
| Pome fruits | 40.5 ± 3.0 (0.0) | 39.5 ± 3.2 (0.0) | 55.5 ± 10.4 (45.4) | 0.026 |
| Stone fruits | 10.0 ± 1.9 (0.0) | 9.9 ± 2.0 (0.0) | 11.9 ± 5.3 (0.0) | 0.564 |
| Berries | 12.8 ± 1.5 (0.0) | 12.9 ± 1.5 (0.0) | 11.2 ± 3.2 (0.0) | 0.339 |
| Wild fruits | 0.0 ± 0.0 (0.0) | 0.0 ± 0.0 (0.0) | 0.0 ± 0.0 (0.0) | 0.180 |
| Raisins | 0.2 ± 0.1 (0.0) | 0.2 ± 0.1 (0.0) | 0.0 ± 0.0 (0.0) | 0.207 |
| Tropical fruits | 40.5 ± 5.1 (0.0) | 40.9 ± 5.4 (0.0) | 35.4 ± 6.6 (15.0) | 0.531 |
| Citrus fruits | 14.9 ± 1.8 (0.0) | 14.0 ± 1.8 (0.0) | 27.7 ± 10.3 (0.0) | 0.200 |
| Canned fruits | 0.8 ± 0.3 (0.0) | 0.8 ± 0.3 (0.0) | 0.6 ± 0.4 (0.0) | 0.537 |
| **Nuts, kernels, and seeds** | 7.3 ± 0.6 (0.0) | 6.8 ± 0.6 (0.0) | 14.0 ± 2.9 (5.6) | 0.007 |
| **Sugars and sweeteners** | 1.4 ± 0.2 (0.0) | 1.4 ± 0.2 (0.0) | 1.3 ± 0.5 (0.0) | 0.975 |
| Sugars | 1.3 ± 0.2 (0.0) | 1.3 ± 0.2 (0.0) | 1.2 ± 0.5 (0.0) | 0.746 |
| Sweeteners | 0.1 ± 0.0 (0.0) | 0.1 ± 0.0 (0.0) | 0.0 ± 0.0 (0.0) | 0.030 |
| **Marmalade, jam, and jelly** | 5.7 ± 0.5 (0.0) | 5.9 ± 0.5 (0.0) | 2.8 ± 0.8 (0.0) | 0.027 |
| **Sweets** | 19.4 ± 1.2 (9.3) | 19.3 ± 1.3 (9.2) | 21.5 ± 5.3 (11.1) | 0.443 |
| Cocoa and cocoa drink powders | 0.1 ± 0.0 (0.0) | 0.1 ± 0.0 (0.0) | 0.1 ± 0.1 (0.0) | 0.853 |
| Chocolates and chocolate products | 7.5 ± 0.6 (0.0) | 7.4 ± 0.6 (0.0) | 9.7 ± 3.6 (0.0) | 0.963 |
| Confectionery and other sweets | 1.9 ± 0.4 (0.0) | 1.9 ± 0.4 (0.0) | 2.0 ± 0.9 (0.0) | 0.485 |
| Ice cream | 6.7 ± 0.9 (0.0) | 6.8 ± 0.9 (0.0) | 6.7 ± 5.1 (0.0) | 0.578 |
| Honey and sweet spreads | 3.2 ± 0.4 (0.0) | 3.2 ± 0.4 (0.0) | 3.0 ± 1.0 (0.0) | 0.498 |
| **Seasonings and other ingredients** | 20.0 ± 1.0 (16.2) | 19.8 ± 1.0 (16.1) | 23.7 ± 3.2 (22.1) | 0.216 |
| **Non-alcoholic beverages** | 1,969.6 ± 61.6 (1,780.2) | 1,954.3 ± 65.0 (1,746.9) | 2,192.1 ± 157.2 (2,156.8) | 0.053 |
| Fruit and vegetable juices | 33.0 ± 4.1 (0.0) | 32.1 ± 4.4 (0.0) | 45.0 ± 9.7 (1.3) | 0.001 |
| Table water | 1,703.8 ± 63.6 (1,488.4) | 1,687.7 ± 66.9 (1,451.6) | 1,936.7 ± 190.3 (1,974.3) | 0.109 |
| Juice spritzer | 62.6 ± 8.8 (0.0) | 62.7 ± 9.1 (0.0) | 61.8 ± 31.2 (0.0) | 0.958 |
| Sodas and lemonades | 112.4 ± 12.2 (0.0) | 113.9 ± 12.7 (0.0) | 91.3 ± 42.8 (0.0) | 0.813 |
| Other non-alcoholic beverages | 28.5 ± 4.9 (0.0) | 28.3 ± 5.1 (0.0) | 31.2 ± 15.1 (0.0) | 0.505 |
| Coffee substitutes | 29.3 ± 6.9 (0.0) | 29.5 ± 7.3 (0.0) | 26.1 ± 10.9 (0.0) | 0.380 |
| **Alcoholic beverages** | 175.9 ± 13.9 (0.0) | 182.8 ± 14.7 (0.2) | 76.1 ± 20.4 (0.0) | 0.018 |
| Spirits | 0.5 ± 0.1 (0.0) | 0.5 ± 0.1 (0.0) | 0.0 ± 0.0 (0.0) | 0.002 |
| Beer | 133.8 ± 13.0 (0.0) | 139.7 ± 13.8 (0.0) | 47.9 ± 18.3 (0.0) | 0.003 |
| Liqueurs and cocktails | 2.8 ± 0.7 (0.0) | 2.9 ± 0.8 (0.0) | 0.3 ± 0.3 (0.0) | 0.747 |
| Wine and sparkling wine | 38.9 ± 3.6 (0.0) | 39.7 ± 3.8 (0.0) | 27.9 ± 10.2 (0.0) | 0.283 |
| **Roasted coffee** | 343.3 ± 15.0 (297.8) | 346.5 ± 15.8 (299.5) | 297.7 ± 42.1 (235.9) | 0.265 |
| **Tea and other infusions** | 299.5 ± 25.7 (0.0) | 287.8 ± 26.5 (0.0) | 469.1 ± 100.3 (221.4) | 0.014 |
| Tea | 113.0 ± 13.3 (0.0) | 111.5 ± 13.9 (0.0) | 133.7 ± 40.5 (0.0) | 0.112 |
| Fruit and herbal tea | 186.6 ± 21.3 (0.0) | 176.3 ± 21.9 (0.0) | 335.4 ± 89.4 (71.8) | 0.012 |
| **Soups and sauces** | 41.6 ± 2.7 (23.4) | 41.5 ± 2.8 (22.8) | 43.1 ± 8.7 (30.3) | 0.840 |
| **Substitute products** | 16.6 ± 2.4 (0.0) | 13.2 ± 2.3 (0.0) | 65.2 ± 15.9 (47.3) | <0.001 |
| Milk substitutes | 13.1 ± 2.2 (0.0) | 10.7 ± 2.1 (0.0) | 47.4 ± 13.7 (0.0) | <0.001 |
| Meat substitutes | 3.5 ± 0.9 (0.0) | 2.5 ± 0.8 (0.0) | 17.8 ± 4.5 (0.0) | <0.001 |
| **Desserts and other sweet dishes** | 3.5 ± 0.9 (0.0) | 2.5 ± 0.8 (0.0) | 17.8 ± 4.5 (0.0) | <0.001 |
| Abbreviations: SE standard error | | | | |
| *^1^*Pooled subsample of individuals self-reporting adherence to either a vegetarian or vegan diet | | | | |
| *^2^*Mean ± SE (Median) | | | | |
| *^3^*Design-based Wilcoxon rank-sum test | | | | |

Supplementary Table 6 Test statistics to the eating motives analyses (n = 1,494). Vegetarians and vegans were pooled as one subsample. Data are weighted to represent the Bavarian population.

|  | **Sex*^3^*** | |  | **Diet type*^3^*** | |
| --- | --- | --- | --- | --- | --- |
| **Eating motive**^4^ | **Test statistic***^1,2^* | **p-value***^1^* |  | **Test statistic***^1,2^* | **p-value***^1^* |
| Liking/Appetite | 0.35 (2.98, 4470.30) | 0.788 |  | 1.02 (2.99, 4480.21 | 0.382 |
| Habits | 1.11 (3.00, 4493.74) | 0.343 |  | 0.07 (2.93, 4395.89) | >0.9 |
| Need and Hunger | 0.76 (2.99, 4482.76) | 0.517 |  | 0.30 (2.93, 4382.92) | 0.823 |
| Health | 6.68 (2.98, 4471.29) | <0.001 |  | 6.21 (2.91, 4358.10) | <0.001 |
| Convenience | 0.60 (2.98, 4463.75) | 0.613 |  | 0.89 (2.95, 4423.28) | 0.446 |
| Pleasure | 0.83 (2.96, 4441.35) | 0.475 |  | 0.32 (2.99, 4484.62) | 0.812 |
| Traditional Eating | 1.25 (2.30, 4492.02) | 0.291 |  | 2.77 (2.89, 4328.84) | 0.042 |
| Natural/Environmental Concerns | 0.06 (2.97, 4450.89) | >0.9 |  | 40.53 (2.91, 4362.34) | <0.001 |
| Sociability | 2.06 (2.99, 4477.19) | 0.104 |  | 3.44 (2.90, 4344.20) | 0.017 |
| Price | 0.13 (2.94, 4401.11) | >0.9 |  | 0.16 (2.93, 4395.39) | >0.9 |
| Visual Appeal | 0.65 (2.98, 4460.96) | 0.580 |  | 0.40 (2.95, 4417.07) | 0.751 |
| Weight Control | 4.31 (2.99, 4476.11) | 0.005 |  | 0.73 (2.96, 4438.24) | 0.535 |
| Affect Regulation | 2.12 (2.83, 4242.39) | 0.099 |  | 0.29 (2.99, 4484.45) | 0.831 |
| Social Norms | 0.97 (2.94, 4411.42) | 0.406 |  | 0.61 (2.80, 4191.08) | 0.595 |
| Social Image | 0.73 (2.88, 4307.88) | 0.526 |  | 0.37 (2.77, 4156.66) | 0.756 |
| *^1^*Chi-squared test with Rao & Scott's second-order correction  *^2^*F (ndf, ddf)  *^3^*Male vs. female; vegetarian/vegan vs. omnivorous | | | | | |
| *^4^*Eating motives are based on Renner et al. (2012) | | | | | |

# Supplementary Figure


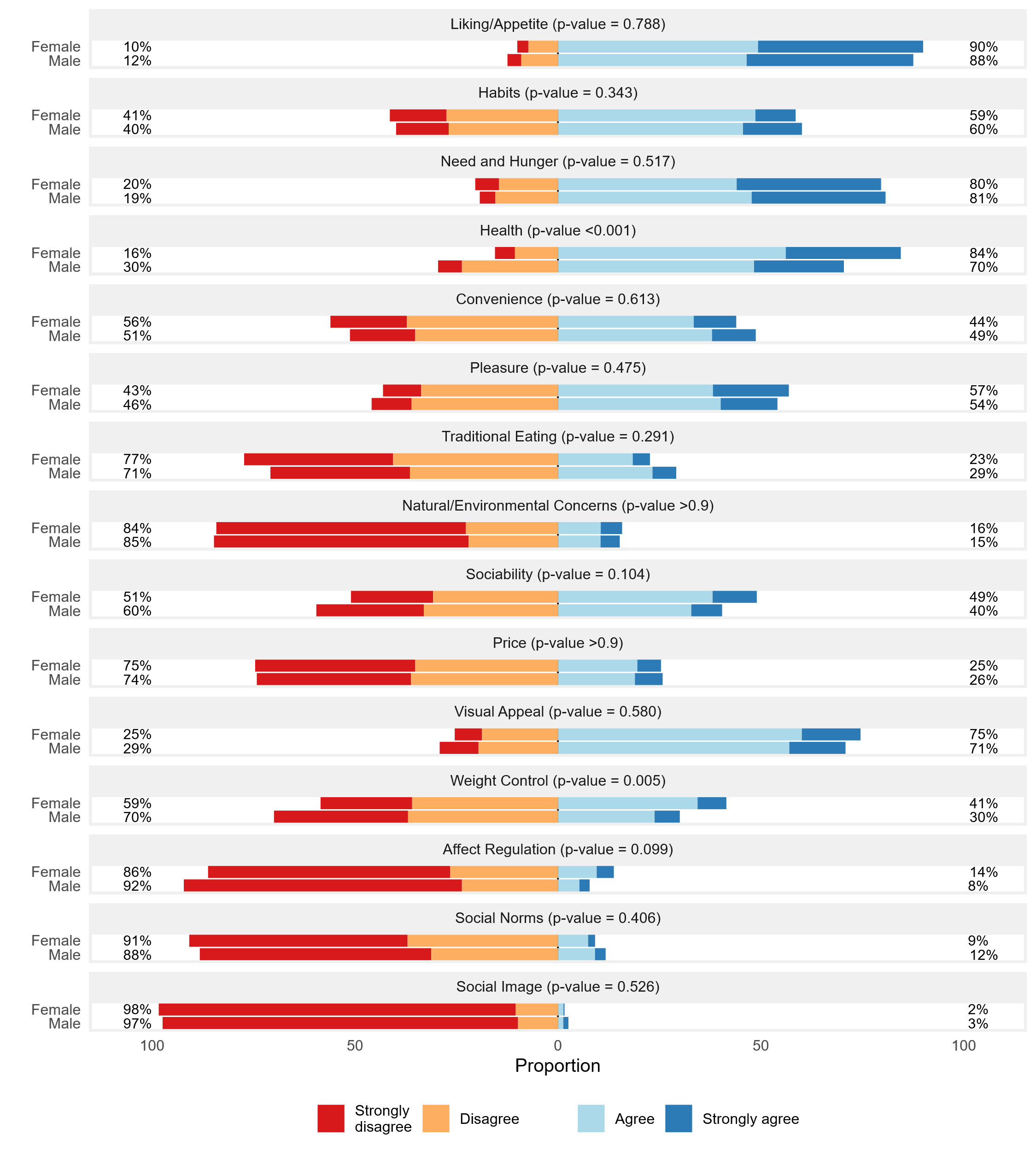


Supplementary Figure 1 Eating motives in Bavaria by sex (n = 1,494). P-values were computed based on chi-squared tests with Rao & Scott's second-order correction. Eating motives are based on Renner et al. (2012). Data are weighted to represent the Bavarian population.

# References

Finger, J. D., J. Tafforeau, L. Gisle, L. Oja, T. Ziese, J. Thelen, G. B. Mensink, and C. Lange. 2015. 'Development of the European Health Interview Survey - Physical Activity Questionnaire (EHIS-PAQ) to monitor physical activity in the European Union', *Archives of Public Health*, 73: 59.

Gerrior, S., W. Juan, and P. Basiotis. 2006. 'An easy approach to calculating estimated energy requirements', *Preventing Chronic Disease*, 3: A129.

Renner, B., G. Sproesser, S. Strohbach, and H. T. Schupp. 2012. 'Why we eat what we eat. The Eating Motivation Survey (TEMS)', *Appetite*, 59: 117-28.

WHO. 2024. 'Obesity and overweight', Accessed 04.11.2024. <https://www.who.int/news-room/fact-sheets/detail/obesity-and-overweight>.
